# Anti-proliferative activity of oral anti-hyperglycemic agents on human vascular smooth muscle cells: thiazolidinediones (glitazones) have enhanced activity under high glucose conditions

**DOI:** 10.1186/1475-2840-6-33

**Published:** 2007-10-28

**Authors:** Peter J Little, Narin Osman, Stephanie T de Dios, Nelly Cemerlang, Mandy Ballinger, Julie Nigro

**Affiliations:** 1 Monash University, Faculty of Medicine, Nursing and Health Sciences, Alfred Hospital, Melbourne, 3004, VIC, Australia; 2 Cell Biology of Diabetes Laboratory, Baker Heart Research Institute, 75 Commercial Rd., Melbourne, 3004, VIC, Australia; 3 Univerisity of Melbourne, Department of Medicine, St. Vincent's Hospital, Melbourne, VIC, 3065, Australia; 4 CSIRO Molecular & Health Technologies, Bayview Avenue, Clayton, VIC 3168, Australia

## Abstract

**Background:**

Inhibition of vascular smooth muscle cell (vSMC) proliferation by oral anti-hyperglycemic agents may have a role to play in the amelioration of vascular disease in diabetes. Thiazolidinediones (TZDs) inhibit vSMC proliferation but it has been reported that they anomalously stimulate [^3^H]-thymidine incorporation. We investigated three TZDs, two biguanides and two sulfonylureas for their ability of inhibit vSMC proliferation. People with diabetes obviously have fluctuating blood glucose levels thus we determined the effect of media glucose concentration on the inhibitory activity of TZDs in a vSMC preparation that grew considerably more rapidly under high glucose conditions. We further explored the mechanisms by which TZDs increase [^3^H]-thymidine incorporation.

**Methods:**

VSMC proliferation was investigated by [^3^H]-thymidine incorporation into DNA and cell counting. Activation and inhibition of thymidine kinase utilized short term [^3^H]-thymidine uptake. Cell cycle events were analyzed by FACS.

**Results:**

VSMC cells grown for 3 days in DMEM with 5% fetal calf serum under low (5 mM glucose) and high (25 mM glucose) increased in number by 2.5 and 4.7 fold, respectively. Rosiglitazone and pioglitazone showed modest but statistically significantly greater inhibitory activity under high versus low glucose conditions (P < 0.05 and P < 0.001, respectively). We confirmed an earlier report that troglitazone (at low concentrations) causes enhanced incorporation of [^3^H]-thymidine into DNA but did not increase cell numbers. Troglitazone inhibited serum mediated thymidine kinase induction in a concentration dependent manner. FACS analysis showed that troglitazone and rosiglitazone but not pioglitazone placed a slightly higher percentage of cells in the S phase of a growing culture. Of the biguanides, metformin had no effect on proliferation assessed as [^3^H]-thymidine incorporation or cell numbers whereas phenformin was inhibitory in both assays albeit at high concentrations. The sulfonylureas chlorpropamide and gliclazide had no inhibitory effect on vSMC proliferation assessed by either [^3^H]-thymidine incorporation or cell numbers.

**Conclusion:**

TZDs but not sulfonylureas nor biguanides (except phenformin at high concentrations) show favorable vascular actions assessed as inhibition of vSMC proliferation. The activity of rosiglitazone and pioglitazone is enhanced under high glucose conditions. These data provide further *in vitro *evidence for the potential efficacy of TZDs in preventing multiple cardiovascular diseases. However, the plethora of potentially beneficial actions of TZDs in cell and animal models have not been reflected in the results of major clinical trials and a greater understanding of these complex drugs is required to delineate their ultimate clinical utility in preventing macrovascular disease in diabetes.

## Background

The role of vascular smooth muscle cell (vSMC) proliferation in vascular disease, particularly atherosclerosis, is controversial and unresolved [1]. However, emerging information is identifying the situations such as post-angioplasty restenosis in people with diabetes in which hyperproliferation is clearly critical in determining the clinical outcome [2]. Although coronary artery by-pass grafting (CABG) was initially the preferred intervention over angioplasty in people with diabetes and coronary artery disease [3] the introduction of coronary artery stents and drug coated stents and possibly supplemented with systemic therapy has raised the possibility that this less invasive treatment may be suitable for this population [4,5]. Although factors such as proteoglycan mediated lipid deposition [6,7] and inflammation [8,9] are clearly important in the process of atherosclerosis and restenosis, in the setting of diabetes vSMC proliferation is clearly critical and thus a target for therapy [2]. As people with diabetes clearly have ongoing hyperglycemia after a clinical intervention for coronary artery disease (CAD), the role of the anti-hyperglycemic therapy in providing a complementary action to prevent vSMC cell proliferation is of potential therapeutic interest [10]. It is further possible that an oral anti-proliferative agent may also be useful as adjunct therapy following vascular intervention even in the absence of diabetes [11].

We have made a direct comparison of the inhibitory activity of the three major classes of oral anti-hyperglycemic agents thiazolidinediones (TZDs) also known as glitazones, biguanides and sulphonylureas towards vSMC proliferation. Further, we used multiple assays to evaluate the mechanism of inhibition and addressed the clinically relevant question of the effect of glucose concentration on the inhibitory activity of the TZDs.

The data shows that only TZDs show appreciable inhibitory activity towards vSMC proliferation amongst currently used oral anti-hyperglycemic agents. Furthermore, under high glucose conditions in which vSMC proliferation is markedly enhanced, the inhibitory potency of the clinical TZDs, rosiglitazone and pioglitazone, is increased not diminished. We also reveal an action of TZDs to stimulate [^3^H]-thymidine incorporation secondary to stimulation of uptake suggesting that other assays of proliferation are more suitable for studies with this class of drug.

## Methods

### Materials

Phenformin, metformin, chlorpropamide, dimethylsulfoxide (DMSO), platelet-derived growth factor (PDGF), Whatman 3 MM chromatography paper and DEAE-Sephacel were obtained from Sigma Aldrich (St Louis, MO). Gliclazide was provided by Institute de Recherches Internationale Servier (France). Troglitazone (Rezulin, Parke-Davis/Warner Lambert), rosiglitazone (Avandia, GlaxoSmithKline) and pioglitazone (Actos, Takeda) were provided as gifts by Parke Davis Pharmaceutical Research (Ann Arbor, MI), GlaxoSmithKline (Boronia, Australia) and Eli Lilly (West Ryde, Australia), respectively. Foetal bovine serum (FBS) was obtained from CSL (Parkville, Australia). [^3^H]-Thymidine and cetylpyridinium chloride (CPC) were from ICN Biomedicals Inc. (Irvine, CA). Scintillation fluid Instagel was from Packard (Groningen, The Netherlands).

### Cell culture preparations

Human vSMCs were isolated using the explant technique from discarded segments of the saphenous veins and internal mammary arteries from a variety of patient donors undergoing surgery at the Alfred Hospital (Melbourne, Australia), the acquisition of which was approved by the Alfred Hospital Ethics committee. Cells from both sources were used for these experiments and in accord with our recent data [12] there were no systematic differences in the results obtained with cells from either vascular origin.

### Analysis of mitogenic response by ^3^H-thymidine incorporation

Cells were seeded at 4.5 × 10^4 ^cells per well in a 24 well plate in DMEM containing 5 mM glucose with 10% FBS for 24 h. Cells were serum deprived for 48 h followed by treatment with control media (0.1% DMSO) or treatment media containing the anti-hyperglycaemic agents in the presence of PDGF (50 ng/ml) and incubated overnight at 37°C in 5% CO_2_. Cells were labelled with [^3^H]-thymidine (1 μCi/ml) or 3 h and assessed for [^3^H] incorporation into newly synthesized DNA as previously described [13].

### Analysis of cell proliferation by assessment of cell number

Human vSMCs in the log growth phase were treated with anti-hyperglycaemic agents (10–300 μM) in the presence of 5% serum for 72 h and assessed for cell number as previously described [12].

### Assessment of thymidine kinase induction by acute [^3^H]-thymidine uptake

Acute [^3^H]-thymidine uptake was assessed as previously described [14]. Briefly, vSMCs were grown to confluency in 24 well plates and serum deprived for 24 h in DMEM (5 mM glucose) and 0.1% FCS. Growth factors and drugs as indicated were added for 18 h. The cells were washed once with DMEM (5 mM glucose) prior to an equilibrating 30 min incubation in DMEM (5 mM glucose). [^3^H]-Thymidine (1 μCi/ml) was added and uptake proceeded for 4 mins at 37°C. At the end of the incubation period, the cells were immediately placed on ice and rapidly washed with ice cold PBS. The radioactivity was released into solution by 1 M NaOH. The amount of [^3^H]-thymidine was quantitated by liquid scintillation counting.

### Cell cycle analysis by Fluorescence Activated Cell Sorting (FACS)

Cells were seeded at 2.5 × 10^5 ^cells per 90 mm plate in duplicate in DMEM containing 5 mM glucose with 10% FBS for 24 h. Cells were serum deprived for 48 h followed by treatment with control media (0.1% DMSO) or treatment media containing the anti-hyperglycaemic agents in the presence of PDGF (50 ng/ml) in the presence of 5% serum and incubated for 72 h at 37°C in 5% CO_2_. For analysis of S phase entry and cells were labelled with 10 μM 5-bromo-2'-deoxyuridine (BrdU, Sigma, St. Louis, MO) for 4 hr at 37°C in the culture medium. BrdU incorporation was detected using FITC-conjugated anti-BrdU (Becton Dickinson) and DNA was stained with propidium iodide (5 μg/ml) 30 min at room temperature in the dark. Flow cytometry and cell cycle analysis was carried out with a FACS Calibur (Becton Dickinson) with 488 nm excitation.

### Statistical analyses

Data are presented as mean ± standard error of the mean (SEM) from 2–5 experiments as indicated in each individual figure. Data were statistically analysed using a Student's t test or 1-way ANOVA.

## Results

### Inhibition of vSMC proliferation by TZDs – dependence of the inhibitory activity of TZDs on the culture media glucose concentration

**Figure 1:**
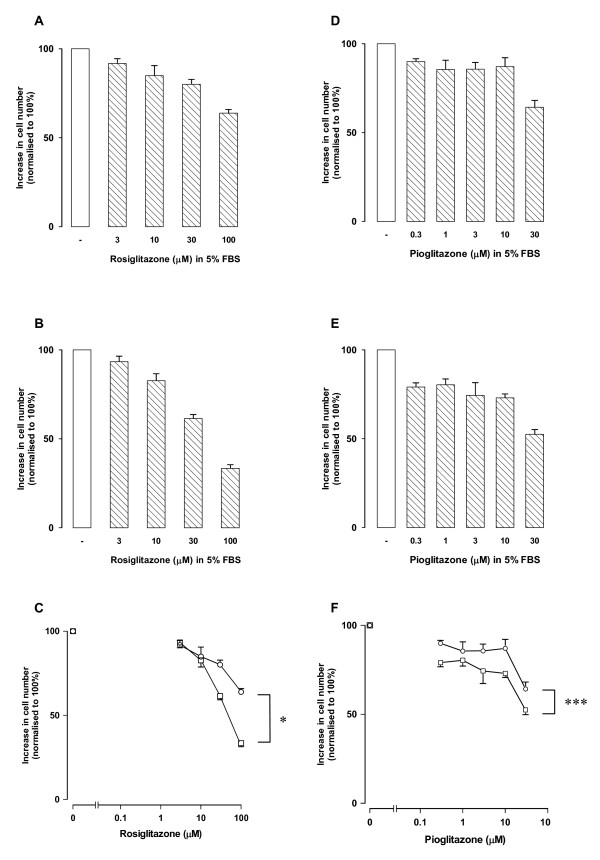
**Effect of media glucose concentration on the inhibitory activity of TZDs**. Panels A and B show the effect of rosiglitazone under low (5 mM) glucose (A) and high glucose (25 mM) and the data shown as a dose response curve (C). Data for pioglitazone is shown in panels D, E and F. Data represent the mean ± SEM from 3 experiments in triplicate *P < 0.05, ***P < 0.001

People with diabetes have blood glucose concentrations that may range at times from 2 to 30 mM and thus the effect of glucose concentration on the actions of anti-hyperglycaemic agents is clinically relevant. We identified a human vSMC preparation that showed enhanced proliferation in response to high glucose media. Under "low" glucose conditions which actually equate to normoglycemia (i.e. DMEM with 5 mM glucose) and in the presence of 5 per cent FBS, cell numbers over 3 days increased from 36,800 ± 3,800 to 91,900 ± 8500 cells/well (mean ± sem, n = 5 experiments in triplicate). Under high glucose conditions (DMEM containing 25 mM glucose) and in the presence of 5 per cent FBS, vSMC numbers increased from 54,400 ± 5500 to 250,500 ± 28,000 cells/well (mean ± sem, n = 5 experiments in triplicate). We used this cell model to assess the inhibitory activity of the two clinical TZDs, rosiglitazone and pioglitazone. Rosiglitazone was evaluated at 3–100 μM and pioglitazone 0.3–30 μM noting that pioglitazone precipitates in the culture media at concentration above 30 μM. The inhibitory effects of rosiglitazone in low and high glucose media were normalised to the control which was set as 100% (Fig. 1A and 1B, respectively). The data is presented as a dose response curve with statistical comparison of the curves (Fig. 1C). The data shows that the inhibitory effect of rosiglitazone is enhanced (P < 0.05) under high glucose condition (Fig. 1C). Similar data for the effect of pioglitazone is shown in (Figs 1D, E and 1F). Pioglitazone showed a greater inhibitory potency (P < 0.001) in high compared to low glucose DMEM.

### Evaluation of the effect of TZDs as assessed by [^3^H]-thymidine incorporation

**Figure 2:**
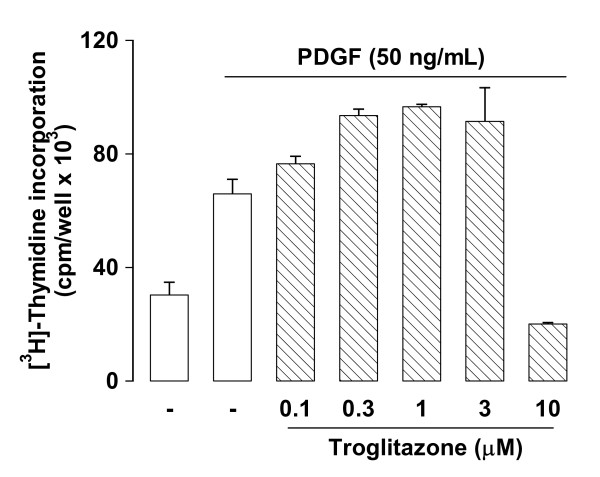
**Effect of troglitazone on PDGF stimulated [^3^H]-thymidine incorporation into DNA**. The experiment was conducted exactly as described in the methods utilising troglitazone (0.1 – 10 μM). The experiment shown is representative of two further experiments in duplicate.

Multiple assays have been used to evaluate the inhibitory effects of TZDs on vSMC proliferation. Takeda et al. [15] have reported that TZDs cause a marked but anomalous increase in [^3^H]-thymidine incorporation in vSMCs and it was further suggested that this is associated with a hyperproliferative response [15]. We investigated the effects of troglitazone, the TZD used by those authors, on the [^3^H]-thymidine incorporation by human vSMCs. Troglitazone had a bi-phasic effect on [^3^H]-thymidine incorporation into DNA with stimulation at low concentrations and inhibition at higher concentrations (Fig. 2). We investigated the mechanism of this increase in [^3^H]-thymidine incorporation which is at variance with the inhibition observed when inhibition of proliferation is assessed by cell counting as we previously described [12].

**Figure 3:**
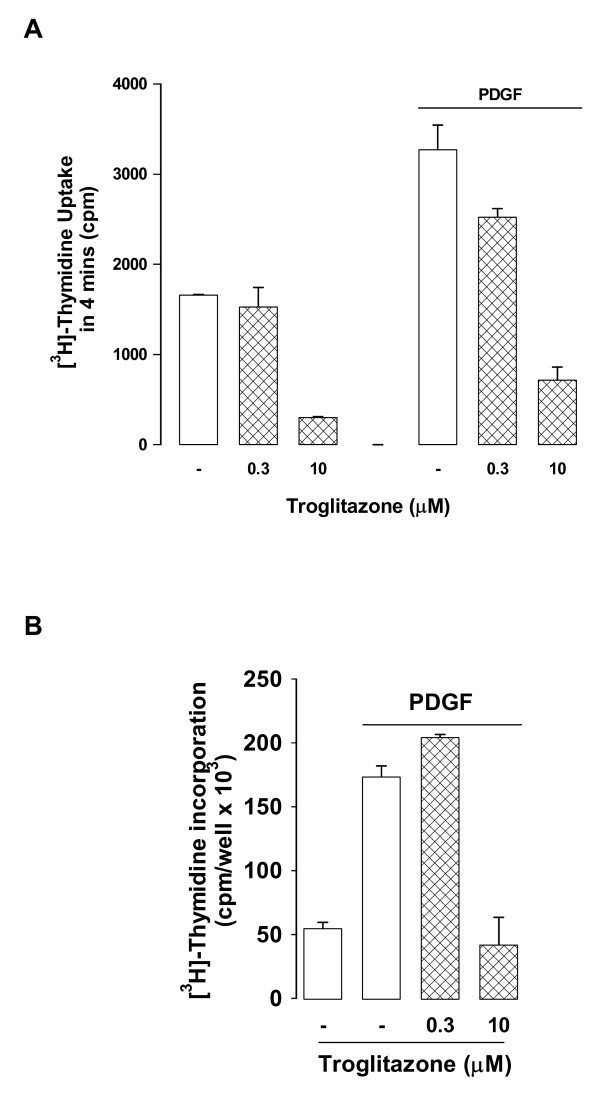
**Effect of TZDs on the induction of thymidine kinase assessed by acute [^3^H]-thymidine uptake and parallel estimation of [^3^H]-thymidine incorporation into DNA**. A. Confluent serum deprived vSMCs were treated with PDGF with and without troglitazone for 20 h then cells were washed free and growth factor and drugs and the acute uptake of [^3^H]-thymidine over 4 min was assessed. The results show the effects of two identical experiments in duplicate. B. Shows concomitant data for routine assay of [^3^H]-thymidine into DNA (see methods for details).

Activation of [^3^H]-thymidine incorporation into DNA of proliferating cells arises from growth factor mediated activation of thymidine kinase activity and enhanced [^3^H]-thymidine uptake into the salvage pathway of thymidylate synthesis which provides adequate precursors for DNA synthesis in vSMC [14]. We hypothesized that troglitazone may have a growth factor like effect (gene induction) on thymidine kinase leading to enhanced uptake and consequently increased incorporation of [^3^H]-thymidine into DNA. Thymidine kinase activity can be assessed as the uptake by the cell of [^3^H]-thymidine over a period of several minutes [14]. We treated vSMCs with low (0.3 μM) and high (10 μM) concentrations of troglitazone which stimulate and inhibit, respectively, [^3^H]-thymidine incorporation into DNA precipitable material (Fig. 2). We undertook the acute (3 min) uptake experiment concomitant with an examination of the effect of troglitazone on incorporation of [^3^H]-thymidine into DNA in a parallel experiment as described above. Troglitazone caused concentration dependent inhibition of the acute uptake of [^3^H]-thymidine (Fig. 3A) with no evidence of stimulation. The parallel experiment for assessing [^3^H]-thymidine incorporation into DNA produced the expected result of enhanced [^3^H]-thymidine incorporation at low concentration of troglitazone (0 – 3 μM) and inhibition of incorporation at high troglitazone concentrations (10 nM) (Fig. 3B).

**Figure 4:**
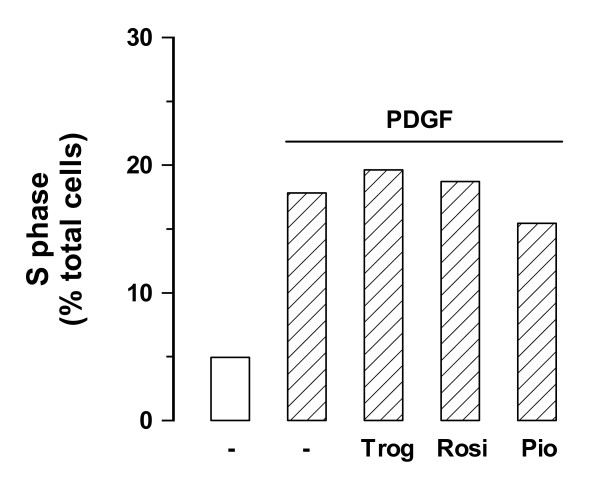
**Assessment of the cell cycle state of human vSMCs following treatment with clinical TZDs**. S phase fraction in cultures treated with TZDs either Troglitazone, Rosiglitazone or Pioglitazone (1 μM each) in the presence of PDGF (50 ng/mL) for 24 h as compared with untreated controls. S phase fraction was analysed by flow cytometry using DNA staining with propidium iodide.

Troglitazone treatment of vSMC increases [^3^H]-thymidine incorporation into newly synthesized DNA which implies that more cells are in the S phase of the cell cycle. We used FACS to investigate the effects of the three TZDs on cell cycle progression in vSMCs. The treatment of vSMCs with troglitazone and rosiglitazone resulted in a small increase in cells in the S phase (Fig. 4). In contrast, treatment of cells with pioglitazone reduced the proportion of cells in the S phase of the cell cycle (Fig. 4). The data indicates that the [^3^H]-thymidine incorporation truly reflects an increased number of cells in the S phase in the presence of rosiglitazone and troglitazone however this does not result in cell cycle progression as no increase in cell numbers is observed [12].

### Effect of biguanides on vSMC proliferation

**Figure 5:**
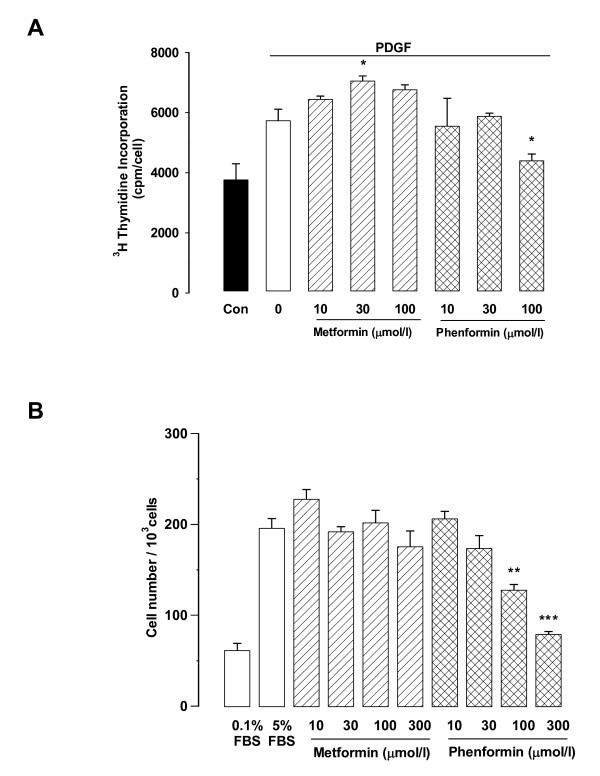
**Effect of metformin and phenformin on vascular smooth muscle cell proliferation assessed by thymidine incorporation and cell counting**. A. Human vSMCs were treated with metformin (10–100 μM) and phenformin (10–100 μM) in the presence of PDGF (50 ng/ml) and assessed for [^3^H]-thymidine incorporation. Data represent the mean ± SEM, *P < 0.05 *vs *PDGF. B. Human vSMCs were treated with metformin (10–300 μM) and phenformin (10–300 μM) in the presence of 5% serum for 3 days and then counted on a Coulter counter. Data represent the mean ± SEM from 2 experiments in triplicate **P < 0.01, ***P < 0.001 *vs *the 5% FBS.

The effects of metformin and phenformin were assessed as described above. Although phenformin has been withdrawn from clinical use, its use also allowed us to assess two compounds in the biguanide class. Metformin had no inhibitory effect on [^3^H]-thymidine incorporation stimulated by PDGF and indeed a small anomalous stimulatory effect at 30 μM that attained statistical significance (Fig. 5A). Phenformin (10, 30, 100 μM) caused a partially concentration dependent inhibitory response with a half maximal inhibitory concentration of approximately 30 μM (Fig. 5A). In terms of vascular pathology the response of most interest is that of changes in cell number. Thus we examined the effect of metformin and phenformin on serum-stimulated increases in cell number. Metformin had no effect on cell numbers but phenformin caused a concentration dependent reduction in cell proliferation with a half maximally inhibitory concentration between 30 and 100 μM (Fig. 5B). There were no apparent toxic effects of phenformin on the cells as assessed by lactate dehydrogenase release and phase contrast microscopy (data not shown).

### Effect of sulphonylureas on vSMC proliferation

We examined the effect of two representative sulfonylureas on the inhibition of cell cycle S-phase [^3^H]-thymidine incorporation into human vSMCs stimulated by PDGF [16]. Neither chlorpropamide (10, 30, 100 μM) nor gliclazide (10, 30, 100 μM) inhibited the ability of PDGF to stimulate [^3^H]-thymidine incorporation even at high concentrations (data not shown). We also assessed the effect of chlorpropamide (3–100 μM) and gliclazide (3–100 μM) to inhibit proliferation assessed by cell counting. Chlorpropamide and gliclazide had no effect on cell proliferation stimulated by 5 per cent fetal bovine serum (FBS) assessed after 3 days proliferation (data not shown).

## Discussion

We have systematically evaluated the effect of the three major classes of oral anti-hyperglycaemic agents for their ability to inhibit vSMC proliferation using the techniques of [^3^H]-thymidine incorporation into DNA and cell counting plus supporting mechanistic studies. We confirmed our earlier report that all three clinically used TZDs cause concentration dependent inhibition of vSMC proliferation assessed by cell counting [12]. Utilizing human vSMCs that showed glucose dependent cell proliferation (accelerated growth under high glucose conditions) we demonstrated that the inhibitory potency of rosiglitazone and pioglitazone was slightly but statistically significantly greater under high glucose conditions. A representative TZD, troglitazone showed an unexpected but previously observed effect to stimulate [^3^H]-thymidine incorporation into DNA at low concentrations with clear and marked inhibition at high concentrations. We investigated the possibility that the stimulation was secondary to induction of thymidine kinase activity and thus acute stimulation of [^3^H]-thymidine uptake but this was not the mechanism. However, cell cycle analysis by FACS confirmed that the increase in [^3^H]-thymidine incorporation correlated with the increase in cells in the S phase of the cell cycle. Two sulfonylureas showed no inhibitory activity in the former assay and were not further investigated. Of the two biguanides only phenformin, which is not now clinically used, showed inhibitory activity and this was expressed in both the assay of [^3^H]-thymidine incorporation into DNA and cell counting. Thus of the clinically relevant oral hypoglycemic agents only the TZDs showed inhibition of vSMC proliferation.

The major clinical implication of these studies relates to the role of vSMC proliferation in cardiovascular disease. The most prominent finding is that the diabetic milieu leads to hyper proliferation of vSMCs such that in people with diabetes proliferation limits the efficacy of vascular interventions due to hyperplasia of vSMCs [2]. Such proliferation also occurs in animal models such as the streptozotocin treated mini pigs in which a clear development of vascular hyperplasia occurs in association with increased expression of inflammatory markers [17]. Thus, there may be a role for choosing an oral hypoglycemic agent with anti-proliferative actions to provide additional potential benefit for people with diabetes. Indeed rosiglitazone has been shown to limit the increase in intimal medial thickening and restenosis in people with diabetes [4,11]. Our demonstration that the anti-proliferative action of TZDs is maintained even enhanced in a high glucose environment favors the use of these agents for glucose lowering in people with diabetes although the overall impact of these agents on cardiovascular disease is the subject of considerable controversy as addressed later [18,19].

The impact of hyperglycemia on the actions of TZDs on cardiovascular cells is an important aspect of the action of anti-hyperglycemic agents, particularly for TZDs which are being considered for wider use in the cardiovascular setting. We are unaware of any reports of the effect of glucose concentration on the cellular action of clinical TZDs in any in vitro setting. There are many examples where high glucose stimulates responses relevant to diabetes [20,21] and it is possible that such stimulation may oppose or sensitize to the actions of TZDs. In our experiments we observed that the activity of pioglitazone and rosiglitazone to inhibit human vSMC proliferation was enhanced under high glucose conditions. It is obviously desirable that the proposed beneficial actions of TZDs not be lost under the hyperglycemia of diabetes.

The unexpected stimulatory effect of troglitazone on [^3^H]-thymidine incorporation [15] does not represent increased cell growth since clearly the cell numbers arising in the presence of the TZD are reduced [12]. The cell cycle has two transition points known as point A in the G_1 _phase and point Q in the G_2 _phase; these are points of hesitation that add a stochastic element to the cell cycle [22]. The data suggest that TZDs, or at least troglitazone, *per se*, can progress vSMC through point A of the cell cycle which leads to S phase DNA synthesis and thus [^3^H]-thymidine incorporation however progression through point Q does not appear to be driven by these agents [22]. We showed that the increase in [^3^H]-thymidine incorporation does not arise from activation of thymidine kinase and thus the mechanism remains unresolved. We have observed in other experiments that TZDs alter glucose metabolism in a manner that perturbs radiolabel incorporation into vSMC proteoglycans (Nigro and Little, unpublished observations) so it is possible that these agents also perturb intracellular nucleotide metabolism in a manner that alters the specific activity of thymidine precursor pools utilized for DNA synthesis. The data indicates that for this drug class, assessment of [^3^H]-thymidine incorporation is not a suitable assay. We have previously demonstrated that the assay of [^3^H]-thymidine incorporation into DNA greatly over estimates the inhibitory effect of the calcium antagonist class of anti-hypertensive drugs [14]. Calcium antagonists acutely block the uptake of [^3^H]-thymidine but because this is not the sole pathway of DNA synthesis, the inhibitory effect of these calcium antagonists is very much smaller when assessed by cell counting [14,23]. Thus, the inhibition of growth factor activity to induce thymidine kinase activity is a superior measure of growth factor activity producing results which reflect the cell number response.

The cardiovascular effects and potential negative outcomes associated with rosiglitazone therapy have been the subject of recent controversy [18]. A meta analysis showed that rosiglitazone therapy was associated with statistically significantly higher rates of myocardial infarction and cardiovascular death [18]. Notably a similar analysis showed that this apparently higher risk of cardiovascular misadventure did not apply to the other clinical TZD, pioglitazone, for which a lower risk of cardiovascular events was calculated [24]. However, an alternative analysis of the rosiglitazone data, an analysis considering for example the role of omitting studies in which there were zero cardiovascular events has shown that the odds ratio for excess cardiovascular events associated with rosiglitazone therapy is much lower and not statistically significant [19] The blood glucose lowering action of TZDs are beneficial in terms of reductions in microvascular complications which are very closely linked to long term estimates of glycaemia [25-27]. However, more than glucose lowering actions are required to manifest beneficial outcomes on cardiovascular disease such as strokes and heart attacks [28]. The lack of human efficacy of TZDs towards cardiovascular disease in initial trials [29] is surprising in view of the overwhelmingly positive occurrence of beneficial actions in cell and animal models for these compounds [30-33]. Nevertheless, human therapeutic experience is the definitive factor and further results from clinical trials will provide information and guidance to the ultimate usefulness of this class of compound [34]. In terms of macrovascular disease the major contributing factors are inflammation, oxidation and the retention of atherogenic lipoproteins by extracellular matrix molecules particularly the proteoglycan, biglycan [35,36]. The role of proteoglycans was demonstrated recently in a human pathology study in coronary arteries in which it was shown that atherosclerosis commences with the deposition lipoproteins associated with the expression of biglycan in the outer layer of the diffuse intimal thickenings [7]. We recently reported on the action of oral anti-hyperglycaemic agents to modify the synthesis and structure of proteoglycans and in accord with the present study it was shown that TZDs are the only group with direct vascular actions [37]. In the present study we observed that the biguanide phenformin inhibited vSMC proliferation and that finding is in accord with our earlier observation that it inhibits protein synthesis in these cells [37]. Metformin appears to be the only non-TZD that shows favourable outcomes on macrovascular disease UKPDS [38] and which may be associated with the important role of insulin resistance in macrovascular disease [39].

## Conclusion

This data supports numerous studies in which TZDs have appreciable direct actions on vSMCs that if expressed in vivo would be beneficial in reducing cardiovascular disease. Our finding that the action of TZDs is enhanced under high glucose conditions is also positive for the use of these compounds in the setting of diabetes. Further understanding is required to match the clinical utility of these agents with the multitude of in vitro and in vivo data which supports a role in the prevention of cardiovascular disease associated with diabetes.

## Abbreviations

CABG: Coronary artery bypass grafting

CAD: Coronary artery disease

DMSO: Dimethylsulfoxide

TZDs: Thiazolidinediones

vSMCs: Vascular smooth muscle cells

## Competing interests

PJL has received honoraria and expenses in relation to presentations at meetings sponsored by GSK. PJL received a GSK post-graduate support award (226/07). JN received travel support from GSK to attend and present related data at scientific meetings.

## Authors' contributions

PJL conceived the study and wrote the manuscript. NO and MB undertook the FACS experiments and assisted with the preparation of the manuscript. SD, NC and JN undertook various experiments.
